# *In Vitro* and *In Vivo* Studies of the Trypanocidal Properties of WRR-483 against *Trypanosoma cruzi*

**DOI:** 10.1371/journal.pntd.0000825

**Published:** 2010-09-14

**Authors:** Yen Ting Chen, Linda S. Brinen, Iain D. Kerr, Elizabeth Hansell, Patricia S. Doyle, James H. McKerrow, William R. Roush

**Affiliations:** 1 Department of Chemistry, The Scripps Research Institute, Scripps Florida, Jupiter, Florida, United States of America; 2 Department of Cellular and Molecular Pharmacology, University of California San Francisco, San Francisco, California, United States of America; 3 Department of Pathology and the Sandler Center for Basic Research in Parasitic Diseases, University of California San Francisco, San Francisco, California, United States of America; New York University School of Medicine, United States of America

## Abstract

**Background:**

Cruzain, the major cysteine protease of *Trypanosoma cruzi*, is an essential enzyme for the parasite life cycle and has been validated as a viable target to treat Chagas' disease. As a proof-of-concept, K11777, a potent inhibitor of cruzain, was found to effectively eliminate *T. cruzi* infection and is currently a clinical candidate for treatment of Chagas' disease.

**Methodology/Principal Findings:**

WRR-483, an analog of K11777, was synthesized and evaluated as an inhibitor of cruzain and against *T. cruzi* proliferation in cell culture. This compound demonstrates good potency against cruzain with sensitivity to pH conditions and high efficacy in the cell culture assay. Furthermore, WRR-483 also eradicates parasite infection in a mouse model of acute Chagas' disease. To determine the atomic-level details of the inhibitor interacting with cruzain, a 1.5 Å crystal structure of the protease in complex with WRR-483 was solved. The structure illustrates that WRR-483 binds covalently to the active site cysteine of the protease in a similar manner as other vinyl sulfone-based inhibitors. Details of the critical interactions within the specificity binding pocket are also reported.

**Conclusions:**

We demonstrate that WRR-483 is an effective cysteine protease inhibitor with trypanocidal activity in cell culture and animal model with comparable efficacy to K11777. Crystallographic evidence confirms that the mode of action is by targeting the active site of cruzain. Taken together, these results suggest that WRR-483 has potential to be developed as a treatment for Chagas' disease.

## Author Summary

Current drugs for Chagas' disease, caused by *Trypanosoma cruzi* infection, are limited in efficacy and are severely toxic. Hence the development of novel chemotherapeutic agents targeting *T. cruzi* infections is an important undertaking. In recent years, there has been considerable interest in cruzain, the major protease in *T. cruzi*, as a target to treat Chagas' disease. Herein, we present the synthesis of WRR-483, a small molecule designed as an irreversible cysteine protease inhibitor, and an assessment of its biological activity against cruzain and *T*. *cruzi* infection. This compound displays pH-dependent affinity for cruzain and highly effective trypanocidal activity in both cell cuture and a mouse model of acute Chagas' disease. The crystal structure of WRR-483 bound to cruzain elucidates the details of inhibitor binding to the enzyme. Based on these results, this inhibitor is a promising compound for the development of therapeutics for Chagas' disease.

## Introduction

American trypanosomiasis, or Chagas' disease, is the third largest parasitic disease burden in the world, and largest in the Western hemisphere. [1] The disease is endemic in Central and South America, and approximately 16 million people are currently afflicted. Patients with Chagas' disease develop flu-like symptoms during the acute stage, followed by gastrointestinal lesions [2] and cardiopathy [3] in the chronic stage. The etiological agent of Chagas' disease is the protozoan parasite, *Trypanosoma cruzi*, which is commonly transmitted to the human host through the bite of the blood sucking triatomine beetle, transfusion of infected blood, or mother-to-child transmission. Nifurtimox and benznidazole, the two drugs used for treatment of Chagas' disease, have significant drawbacks, as they are at best moderately effective in the chronic stages of the infection and cause severe side effects. [4], [5] Hence, the development of novel therapeutics to effectively treat Chagas' disease is essential.

**Figure 1:**
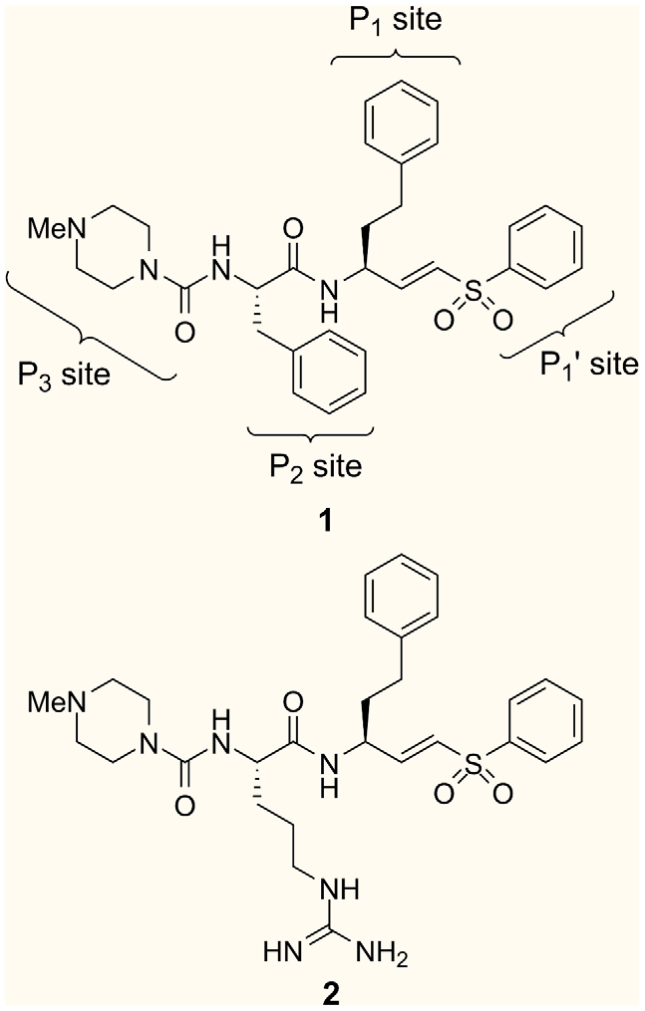
Structure of vinyl sulfone inhibitors, K11777 (1) and WRR-483 (2). The P_1_ –P_3_ subsites of K11777 are labeled.

The major cysteine protease of *T. cruzi*, cruzain, is an attractive target for the development of trypanocidal agents. Cruzain is expressed throughout the parasite life cycle and plays important roles in the survival of the organism, including immunoevasion, acquisition of nutrients, and parasite differentiation. [6] In addition, the lack of redundancy of this enzyme makes the parasites vulnerable to cruzain inhibition. In recent years, K11777 (**1**, Figure 1), a selective cruzain inhibitor, has been demonstrated to eradicate *T. cruzi* infection in cell culture, mouse, and dog models. [7], [8], [9] These studies prove that cysteine protease inhibitors could serve as a viable agent for chemotherapeutic intervention.

X-ray crystal structures of cruzain in complex with reversible [10], [11]and irreversible inhibitors [12], [13], [14], [15], [16], [17] have been reported, and the overall folding pattern and structure of the active site is highly homologous to papain. Seven substrate binding sites, four (S_4_, S_3_, S_2_, and S_1_) on the acyl side and three (S_1_′, S_2_′, and S_3_′) on the amino side of the scissile bond, form a cleft between two structural domains of the enzyme. The catalytic triad of Cys25, His159, and Asn175, as well as the highly conserved Trp177, is contained within this cleft. Like most other papain-like cysteine proteases, the interaction of the S_2_ site of the enzyme with the complementary P_2_ residue is the key specificity determining factor. Cruzain is able to accommodate phenylalanine or arginine in the P_2_ position of the ligand due to the presence of Glu208 (cruzain numbering) found at the base of the S_2_ pocket, which can form a salt bridge with the positively charged arginine side chain. [13],[18]

A variety of cysteine protease inhibitors have been reported in the literature. [19],[20],[21],[22] In one of our group's strategies in designing parasitic cysteine protease inhibitors, we have developed peptidyl vinyl sulfones based on the pioneering work by Hanzlik [23]and Palmer. [24]The vinyl sulfone warhead acts as a Michael acceptor for the active site Cys25, while the sulfone unit and the peptide framework provide several hydrogen bond acceptors that interact favorably with complementary residues in the active site.[14] In our earlier reports, we investigated the structure-activity relationship of these inhibitors with variations on the vinyl sulfone substituent, the P_1_ side chain, and the P_3_ group to generate a series of highly potent cruzain inhibitors. [25] Further studies led to the identification of compounds that effectively disrupted *T. cruzi* infection in cell culture assays; [26]however, most of these compounds proved to be too weak to be effective drugs in the *in vivo* mouse model. To date, all of our compounds contain a hydrophobic group at the P_2_ site. Herein, we report the synthesis of WRR-483 (**2**), the arginine variant of K11777, its remarkable biological properties, and a crystal structure of WRR-483 bound to cruzain.

## Methods

### Chemistry: General methods

All reaction solvents were of reagent grade and used as received. Tetrahydrofuran, dichloromethane, diethyl ether, and toluene were purified by passing through a solvent column composed of activated A-1 alumina. Unless indicated otherwise, all reactions were conducted under an atmosphere of nitrogen using flame-dried or oven-dried (170°C) glassware. Proton nuclear magnetic resonance (^1^H NMR) spectra and carbon-13 (^13^C) NMR spectra were recorded on commercially available NMR spectrometers at 400 MHz and 100 MHz, respectively. The proton signal for residual, non-deuterated solvent (δ 7.26 ppm for CHCl_3_, δ 2.50 ppm for DMSO, and δ 3.31 ppm for MeOD) was used as an internal reference for ^1^H NMR spectra. For ^13^C NMR spectra, chemical shifts are reported relative to the δ 77.0 ppm resonance of CDCl_3_, δ 23.0 ppm for DMSO, or the δ 49.0 ppm resonance of MeOD. Coupling constants are reported in Hertz (Hz). Mass spectra were recorded at the University of Michigan Mass Spectrometry Laboratory.

Analytical thin layer chromatography (TLC) was performed on Kieselgel 60 F_254_ glass plates pre-coated with a 0.25 mm thickness of silica gel. The TLC plates were visualized with UV light and/or by staining with either Hannesian solution (ceric sulfate and ammonium molybdate in aqueous sulfuric acid) or permanganate solution (potassium permanganate in aqueous sodium hydroxide). Column chromatography was generally performed using Kieselgel 60 (230–400 mesh) silica gel, typically using a 50-100:1 weight ratio of silica gel to crude product.

#### (S)-Benzyl 2-amino-5-(3-(2,2,4,6,7-pentamethyl-2,3-dihydrobenzofuran-5-ylsulfonyl)guanidino)pentanoate (4)

To a 0°C solution of *Nα*-(9-fluorenylmethoxycarbonyl)-*Nω*-(2,2,4,6,7-pentamethyldihydrobezofuran-5-sulfonyl)-L-arginine (**3**, Fmoc-Arg(Pbf)-OH, 0.636 g, 0.980 mmol), benzyl alcohol (0.112 mL, 1.08 mmol), *N*-methylmorpholine (0.120 mL, 1.09 mmol) and 4-dimethylaminopyridine (DMAP, 10 mg) in dichloromethane (CH_2_Cl_2_, 10 mL) was added 1-ethyl-3-(3′-dimethylaminopropyl)carbodiimide hydrochloride (EDC, 0.223 g, 1.16 mmol). The resulting mixture was stirred for 1 h at 0°C then 12 h at room temperature. The solvent was removed by rotary evaporation, and the residue was treated with ethyl acetate (50 mL). The organic layer was washed with a saturated solution of aqueous sodium bicarbonate (20 mL), brine (20 mL), dried over Na_2_SO_4_, filtered, and concentrated to dryness to yield the crude benzyl ester as a white foam. A solution of 20% piperidine in CH_2_Cl_2_ dichloromethane (4 mL) was added to the crude benzyl ester and the mixture was stirred for 1 h at room temperature. The solvent was removed by rotary evaporation and the residue was purified by flash column chromatography (5–9% methanol in CH_2_Cl_2_) to give compound **4** (0.458 g, 0.886 mmol, 90% overall) as colorless foam: ^1^H NMR (400 MHz, CDCl_3_) δ 7.34 (m, 5 H), 6.26 (br s, 1 H), 6.20 (s, 2 H), 5.12 (s, 2 H), 3.48 (m, 1 H), 3.14 (m, 2 H), 2.93 (s, 2 H), 2.56 (s, 3 H), 2.50 (s, 3 H), 2.08 (s, 3 H), 1.78 (m, 1 H), 1.72 (s, 2 H), 1.58 (m, 3 H), 1.44 (s, 6H); ^13^C NMR (100 MHz, CDCl_3_) δ 175.6, 158.9, 156.3, 138.5, 135.7, 133.2, 132.5, 128.9, 128.7, 128.6, 124.8, 117.7, 86.6, 67.1, 54.2, 43.4, 41.0, 28.8, 25.8, 19.5, 18.1, 12.7; HRMS (ES+) *m/z* for C_26_H_36_N_4_NaO_5_S [M+Na]^+^ calcd 539.2304, found 539.2304.

#### (S)-Benzyl 2-(4-methylpiperazine-1-carboxamido)-5-(3-(2,2,4,6,7-pentamethyl-2,3-dihydrobenzofuran-5-ylsulfonyl)guanidino)pentanoate (5)

A solution of amine **4** (0.809 g, 1.57 mmol) in CH_2_Cl_2_ (25 mL) was vigorously stirred for 30 min at 0°C with a saturated solution of aqueous sodium bicarbonate (25 mL). A solution of triphosgene (0.155 g, 0.52 mmol) in CH_2_Cl_2_ (2 mL) was added and the reaction mixture was stirred at 0°C for another 30 min. The layers were separated and the aqueous layer was further extracted three times with CH_2_Cl_2_ (10 mL). The combined organic layers were dried over Na_2_SO_4_, filtered and cooled to 0°C. *N*-Methylpiperazine (0.156 g, 1.56 mmol) was added to the solution. This mixture was stirred overnight and then concentrated to dryness. Purification of the residue by flash column chromatography (7–12.5% methanol in CH_2_Cl_2_) gave 0.939 g (93% overall) of urea **5** as colorless foam: ^1^H NMR (400 MHz, CDCl_3_) δ 7.33 (m, 5 H), 6.32 (br s, 1 H), 6.23 (br s, 2 H), 5.37 (d, *J* = 7.2 Hz, 2 H), 5.20 (d, *J* = 12.3 Hz, 1 H), 5.15 (d, *J* = 12 Hz, 1 H), 4.50 (m, 1 H), 3.38 (app t, *J* = 4.8 Hz, 4 H), 3.29 (br s, 1 H), 3.14 (br s, 1 H), 2.93 (s, 2 H), 2.55 (s, 3 H), 2.49 (s, 3 H), 2.33 (app t, *J* = 4.8 Hz, 4 H), 2.27 (s, 3 H), 2.07 (s, 3 H), 1.80–1.91 (m, 1 H), 1.57–1.69 (m, 3 H), 1.44 (s, 6H); ^13^C NMR (100 MHz, CDCl_3_) δ 173.5, 159.0, 157.7, 156.5, 138.7, 135.5, 133.5, 132.6, 129.0 (two peaks), 128.7, 124.9, 117.8, 86.7, 67.8, 54.9, 46.4, 44.1, 43.6, 41.1, 31.6, 29.0, 25.3, 19.6, 18.2, 12.8; HRMS (ES+) *m/z* for C_32_H_46_N_6_NaO_6_S [M+Na]^+^ calcd 665.3097, found 665.3099.

#### (S)-2-(4-Methylpiperazine-1-carboxamido)-5-(3-(2,2,4,6,7-pentamethyl-2,3-dihydrobenzofuran-5-ylsulfonyl)guanidino)pentanoic acid (6)

Urea **5** (1.65 g, 2.57 mmol) was dissolved in a mixture of ethyl acetate (4 mL) and methanol (16 mL). To this solution was added 10% palladium on carbon (0.25 g) and the reaction was stirred under a hydrogen atmosphere using a balloon filled with hydrogen gas for 20 h at room temperature. After removal of the catalyst by filtration through a pad of CELITE™, the filtrate was concentrated *in vacuo* to afford 1.31 g (92%) of acid **6** as a colorless solid: ^1^H NMR (400 MHz, MeOD-*d*_4_) δ 4.17 (dd, *J* = 8.0, 4.8 Hz, 1 H), 3.62 (m, 4 H), 3.17 (m, 2 H), 2.99 (m, 6 H), 2.70 (s, 3 H), 2.57 (s, 3 H), 2.51 (s, 3 H), 2.08 (s, 3 H), 1.81–1.88 (m, 1 H), 1.65–1.70 (m, 1 H), 1.57–1.63 (m, 2 H), 1.45 (s, 6 H); ^13^C NMR (100 MHz, MeOD-*d*_4_) δ 178.6, 159.9, 159.1, 158.2, 139.4, 133.5, 118.5, 87.7, 56.3, 54.7, 44.5, 44.0, 41.7, 30.8, 28.7, 27.1, 19.6, 18.4, 12.5; HRMS (ES+) *m/z* for C_25_H_40_N_6_NaO_6_S [M+Na]^+^ calcd 575.2628, found 575.2615.

#### 4-Methyl-N-((S)-1-oxo-5-(3-(2,2,4,6,7-pentamethyl-2,3-dihydrobenzofuran-5-ylsulfonyl)guanidino)-1-((S,E)-5-phenyl-1-(phenylsulfonyl)pent-1-en-3-ylamino)pentan-2-yl)piperazine-1-carboxamide (8)

Vinyl sulfone **7** (0.864 g, 2.15 mmol) was dissolved in a solution of 33% trifluoroacetic acid in CH_2_Cl_2_ (7.5 mL) and the mixture was stirred in an ice bath for 2 h. The solvent was removed, and the excess trifluoroacetic acid was removed by repeated evaporation with toluene *in vacuo*. The crude amine (as the TFA salt) was treated with CH_2_Cl_2_ (10 mL) and enough dimethylformamide (DMF, *ca.* 2 mL) to give a clear solution. To this solution were added acid **6** (1.19 g, 2.16 mmol), N-hydroxybenzotriazole (HOBT, 0.363 g, 2.37 mmol), *N*-methylmorpholine (0.474 mL, 4.32 mmol), and 1-ethyl-3-(3′-dimethylaminopropyl)carbodiimide hydrochloride (EDC, 0.454 g, 2.37mmol). The resulting mixture was stirred at 0°C then warmed to room temperature over 11 h. After removal of solvent by rotary evaporation, the residue was dissolved in ethyl acetate (80 mL) and extracted with saturated aqueous solution of sodium bicarbonate (20 mL). The layers were separated and the organic layer was washed with brine (20 mL), dried over sodium sulfate, filtered, and the solvent was removed *in vacuo*. Purification of the crude product by flash column chromatography (11–14% methanol in CH_2_Cl_2_) provided 1.56 g (84%) of vinyl sulfone **8** as a colorless solid: ^1^H NMR (400 MHz, DMSO-d*_6_*) δ 7.99 (d, *J* = 8.4 Hz, 1H), 7.82 (d, *J* = 7.2 Hz, 2H), 7.70 (t, *J* = 7.4 Hz, 1H), 7.62 (t, *J* = 7.4 Hz, 2H), 7.25 (t, *J* = 7.6 Hz, 2H), 7.16 (t, *J* = 7.0 Hz, 3H) 6.87 (dd, *J* = 15.2, 4.8 Hz, 1H), 6.70 (dd, *J* = 15.2, 1.6 Hz, 1 H), 6.60–6.90 (br s, 1 H), 6.46 (d, *J* = 7.6 Hz, 1 H), 6.38 (br s, 1 H), 4.45 (m, 1 H), 3.99 (m, 1 H), 3.29 (m, 4 H), 3.02 (m, 2 H), 2.95 (s, 2 H), 2.54–2.61 (m, 1 H), 2.48 (s, 3 H), 2.43 (s, 3 H), 2.40–2.51 (m, 1 H), 2.21 (m, 4 H), 2.14 (s, 3 H), 2.00 (s, 3 H), 1.89–1.96 (m, 1 H), 1.72–1.81 (m, 1 H), 1.54–1.64 (m, 2 H), 1.40 (s, 6H), 1.31–1.48 (m, 2 H); ^13^C NMR (100 MHz, DMSO-*d*_6_) δ 172.8, 157.4, 157.3, 156.0, 147.1, 141.1, 137.3, 133.6, 131.4, 12.8, 129.6, 128.3, 127.0, 125.8, 124.3, 116.2, 86.3, 54.5, 54.4, 48.5, 45.7, 43.4, 42.4, 40.1, 39.9, 39.7, 39.5, 39.3, 39.1, 38.9, 34.5, 31.3, 28.8, 28.3, 18.9, 18.5, 17.6, 12.3; HRMS (ES+) *m/z* for C_42_H_58_N_7_O_7_S [M+H]^+^ calcd 836.3834, found 836.3869.

#### WRR-483 (2)

Trifluoroacetic acid (3 mL) was added to a solution of the protected vinyl sulfone **8** (0.4056 g, 0.485 mmol) in CH_2_Cl_2_ (1 mL) at 0°C and the reaction mixture was stirred for 4.5 h. The solvent was removed under reduced pressure and excess trifluoroacetic acid was removed by repeated evaporation with toluene *in vacuo*. The crude product was triturated in Et_2_O and the solvent was decanted. The solid residue was dissolved in 0.2 N HCl (15 mL) and washed four times with ethyl acetate (10 mL). Water was removed *in vacuo* and the resulting oil was triturated with acetonitrile to give 0.273 g (86%) of WRR-483 (**2**) as a colorless solid (HCl salt): ^1^H NMR (400 MHz, MeOD-*d*_4_) δ 7.88 (d, *J* = 7.2 Hz, 2 H), 7.70 (t, *J* = 7.6 Hz, 1 H); 7.61 (t, *J* = 7.6 Hz, 2 H), 7.24 (t, *J* = 7.6 Hz, 2 H), 7.16 (m, 3 H), 6.89 (dd, *J* = 15.0, 5.0 Hz, 1 H), 6.66 (dd, *J* = 14.8, 1.6 Hz, 1 H), 4.55 (m, 1 H), 4.31 (br s, 2 H), 4.14 (dd, *J* = 8.0, 6.4 Hz, 1 H), 3.50 (br s, 2 H), 3.22 (t, *J* = 6.4 Hz, 4 H), 3.10 (br s, 2 H), 2.93 (s, 3 H), 2.71 (ddd, *J* = 13.6, 8.8, 5.6 Hz, 1 H), 2.61 (m, 1 H), 1.96 (m, 2 H), 1.83 (m, 2 H), 1.75 (m, 1 H), 1.66 (m, 1 H); ^13^C NMR (100 MHz, MeOD-*d*_4_) δ 175.3, 158.8, 158.6, 147.6, 142.2, 141.8, 134.9, 132.2, 130.6, 129.6, 129.5, 128.7, 127.2, 56.9, 4.4, 50.7, 43.7, 42.5, 42.1, 36.1, 33.2, 30.1, 26.7; HRMS (ES+) *m/z* for C_29_H_42_N_7_O_4_S [M+H]^+^ calcd 584.3019, found 584.3026.

### Enzyme assays

Cruzain[27],[28], rhodesain [29], and tbcatB [30] were recombinantly expressed as described previously. TbcatB assays were performed as described previously.[31] Cruzain (2 nM) or rhodesain (3 nM) was incubated with 0.5 to 10 µM inhibitor concentration in 100 mM sodium acetate at pH 5.5, containing 5 mM DTT (buffer A) for 5 min at room temperature. Then buffer A containing Z-Phe-Arg-AMC (Bachem, *K*_M_ = 1 µM) was added to enzyme inhibitor to give 20 µM substrate concentration in 200 µL, and the increase in fluorescence (excitation at 355 nm and emission at 460 nm) was followed with an automated microtiter plate spectrofluorometer (Molecular Devices, Flex station). Inhibitor stock solutions were prepared at 20 mM in DMSO, and serial dilutions were made in DMSO (0.7% DMSO in assay). Controls were performed using enzyme alone and enzyme with DMSO. IC_50_ values were determined graphically using inhibitor concentrations in the linear portion of a plot of inhibition versus log[I] (seven concentrations tested with at least two in the linear range).

For pH dependence studies, cruzain was tested at 4 nM in 5 µM Z-Phe-Arg-AMC in 0.15 M citrate phosphate buffer at pH 5.5 and pH 8.0 with 5 mM DTT, and 0.01% Triton-X 100. Enzyme was added to wells of a 96-well microtiter plate containing substrate and inhibitor diluted in DMSO (0.5% final concentration), or DMSO control. Final inhibitor concentration ranged from 0.01 µM to 10 µM. Experiments were done in triplicate. Assays were run at 25°C in an automated microtiter plate spectrofluorometer, with robotic delivery of enzyme and readings every 1.52 seconds throughout the assay, before and after enzyme addition.

Inhibitor dilutions which produce simple exponential progress curves over a wide range of *k*_obs_, were used to determine kinetic parameters. The value of *k*_obs_, the rate constant for loss of enzyme activity, was determined from an equation for pseudo first order dynamics using Prism 4.0 (GraphPad). When *k*_obs_ varied linearly with inhibitor concentration, *k*_ass_ was determined by linear regression analysis. If the variation was hyperbolic, indicating saturation inhibition kinetics, *k*_inact_ and *K*_i_ were determined from an equation describing a two-step irreversible inhibitor mechanism [*k*_obs_ = *k*_inact_ [I]_o_/([I]_o_+*K*i* (1+[S]_o_/*K*_M_))] and non-linear regression analysis Prism 4.0.[32] All values were corrected for substrate concentration.

### *T. cruzi* cell culture assay

CA-I/72 *T. cruzi* parasites were isolated from a chronic Chagasic patient, cloned, and maintained as previously described.[33]

For growth inhibition studies, J774 macrophages cultured in RPMI-1640 medium with 5% heat inactivated fetal calf serum (FCS) were irradiated (9000 rad) to arrest the cell cycle and plated onto 12-well tissue culture plates for 24 h at 37°C. After infection with 10^5^ *T. cruzi* trypomastigotes per well for 2 hours, monolayers were washed with RPMI medium and treated with the inhibitors at 10 µM in RPMI medium (triplicate wells per inhibitor). Inhibitor stocks were made to 10 mM in DMSO and diluted prior to use. All assays include untreated, K11777-treated, and uninfected macrophage controls. Fresh medium with or without inhibitor was replaced every 48 h and inhibitor efficacy was monitored daily. Survival time was defined as the time before the cell monolayer was destroyed by the infection. Under these culture conditions, *T. cruzi* completed the intracellular cycle in 6 days in untreated controls. Treatment duration was up to 27 days as such regime results in cure of macrophages treated with 10 µM K11777 (positive control). Macrophages were subsequently cultured in normal medium for up to 40 days to elucidate if the effective inhibitors were trypanocidal (cure host macrophages) or trypanostatic (delay intracellular cycle of the parasite).

For dose-response studies, bovine embryo skeletal muscle (BESM) cells were infected with *T. cruzi* as previously described with minor modifications.[34] Briefly, 150 µL of RPMI medium containing 1000 BESM cells were seeded per well in a 96-well plate and incubated for 4 h at 37°C to allow cell attachment. Monolayers were then infected with 1000 trypomastigotes/well of CA-I/72 *T. cruzi* for 2 h at 37°C. Cells were washed once with 200 µL of sterile PBS and medium was replaced with WRR-483 at the following concentrations: 20, 10, 7.5, 5, 2.5, 1.25, 0.6 and 0.3 µM. Cultures were then incubated for 72 h at 37°C in a humidified atmosphere with 5% CO_2_. Cultures were next fixed in 4% fresh paraformaldehyde in PBS, stained with DAPI, and counted under a fluorescence microscope (400×). Mean numbers of parasites per cell (±SE) were calculated as previously reported (n = 3–4 per concentration). Controls consisted of untreated wells and cultures similarly treated with 10 µM K777 and 0.1 µM posaconazole.

### *In vivo* studies

#### Ethics statement

These studies were performed in strict accordance with the recommendations in the Guide for the University of California San Francisco Institutional Animal Care and Use Committee. The protocol was approved by the Committee on the Ethics of Animal Experiments of the University of California San Francisco (Permit Number: AN080380-02). All surgery was performed under sodium pentobarbital anesthesia, and all efforts were made to minimize suffering.

Three to four week old female C3H mice weighing *ca.* 20 g were used. The animals were separated into five lots of 5 mice per cage and infected with 10^6^ tissue-culture derived trypomastigotes of the myotropic *T. cruzi* CA-I/72 cloned stock. The mice were treated with 100 mg compound/kg of body weight in 100 µL of solution (30–40% DMSO: 60–70% sterile distilled water) intraperitoneally twice a day. Treatment was initiated 12 h post infection and continued until cure, death of the animals, or for a total of 20 days. Controls included one lot of infected, untreated animals and another lot of infected animals treated daily with 100 mg K11777/kg weight in two daily doses. Parasitemias was determined at the end of the experiment when animals were euthanized. Tissues processed for histopathology include skeletal muscle, heart, liver, spleen, and colon. Blood (5–50 µL) was used for hemocultures. Hemocultures were considered negative if no parasites were observed for 60 days. Treatment was considered effective if life expectancy is increased in treated animals compared to untreated controls, symptoms of acute Chagasic infection were absent, and histopathological observation showed normal tissues and no parasites. Compounds were considered toxic if life expectancy is lower than untreated controls (negative values). If necessary, PCR of blood and tissues was performed to confirm effectiveness of treatment and/or cure of treated animals. Results were expressed as survival (days) of treated animals.

### Preparation, purification and crystallization of cruzain bound to WRR-483

Cruzain was expressed and purified as described previously.[27] Activated cruzain was incubated overnight with molar excess amounts of inhibitor dissolved in DMSO. This prevented further proteolytic activity and lack of activity was confirmed *via* fluorometric assay against the substrate Z-Phe-Arg-AMC (Bachem, *K*_M_ = 1 µM). After passage over a MonoQ column, fractions containing pure inhibited cruzain were concentrated, to a final concentration of 10 mg/mL, with a Viva-Spin (Viva Science) column (MWCO 15 kDa). Simultaneous with concentration, buffer exchange to 2 mM bis-tris at pH 5.8 was performed. Crystals of maximum size were obtained after approximately 1 week *via* the hanging drop method, from a precipitating agent of 1.26 M (NH_4_)_2_SO_4_, 0.2 M LiSO_4_, at pH 6.0. Crystals were flash-cooled in liquid nitrogen after a 5 second soak in 20% ethylene glycol and loaded into a SAM (Stanford Auto Mounter) cassette for crystal screening.[35]

### Data collection, structure solution and crystallographic refinement

All diffraction data were collected at the Stanford Synchrotron Radiation Laboratory (SSRL), beamline 9-1, using monochromatic radiation of 0.98 Å, after selecting an optimal crystal from screening performed with the robotic SAM system. An ADSC Quantum 315 3x3 CCD array detector was used with low temperature conditions of 103 K at the crystal position. Data processing was completed with MOSFLM [36] and SCALA. The structure was solved *via* molecular replacement using the MOLREP program of the CCP4 suite [37] with a model derived from cruzain bound to a different vinyl-sulfone containing inhibitor (PDB ID 1F2A), with inhibitor and water molecules removed from the search model. The topmost solution contained two unique monomers related by an NCS two-fold axis of symmetry. The solution was 82.2 σ above noise level, with an R_factor_ of 0.493. Iterative rounds of manual model building and refinement were completed with COOT and Refmac5 with isotropic temperature factors. The inhibitor molecule was manually placed and fit to electron density using COOT. Clear and representative density for the entirety of both inhibitor molecules in the asymmetric unit was observed at better than 1.5 σ above the noise level. Water molecules were placed with COOT and manually assessed. Molecules of the cryoprotectant ethylene glycol and the crystallization precipitant ammonium sulfate were also discernable in final electron density maps of this structure and were placed using COOT and refined with Refmac5. All statistics for data collection, structure solution and refinement are given in Table 1. The coordinates and observed structure factor amplitudes for the refined structure have been deposited in the Protein Data Bank under accession code 3LXS.

**Table 1:** Crystallographic parameters: data collection and refinement statistics (for high resolution bin, statistics are given in parentheses).

**Data Collection**	
Resolution (Å)	1.50
Space group	C2
Unit cell parameters	
a (Å)	134.35
b (Å)	38.14
c (Å)	95.16
β (°)	114.36
Wavelength (Å)	0.98
Temperature (K)	103
Total number reflections	374500
Total unique reflections	70666
Completeness	99.2 (99.7)
Redundancy	5.3 (5.4)
R_sym_	0.057 (0.182)
*I/σI*	19.3 (8.9)
**Refinement**	
Resolution range (Å)	45.31 – 1.50 (1.54 -1.50)
R_factor_	0.122 (0.121)
R_free_	0.158 (0.181)
Average B factor (Å^2^)	
ethylene glycol	26.50
inhibitor	13.08
protein	10.01
sulfate	32.60
water	24.00
R.m.s deviation from ideal	
Bond lengths (Å)	0.017
Bond angles (°)	1.76
Ramachandran analysis (MolProbity)	
Residues in favored regions (%)	97.9
Residues in allowed regions (%)	100
Residues in disallowed regions (%)	0
Molprobity	
Score	2.48
Percentile	99%
PDB ID	3LXS

## Results

### Synthesis of WRR-483 (2)

**Figure 2:**
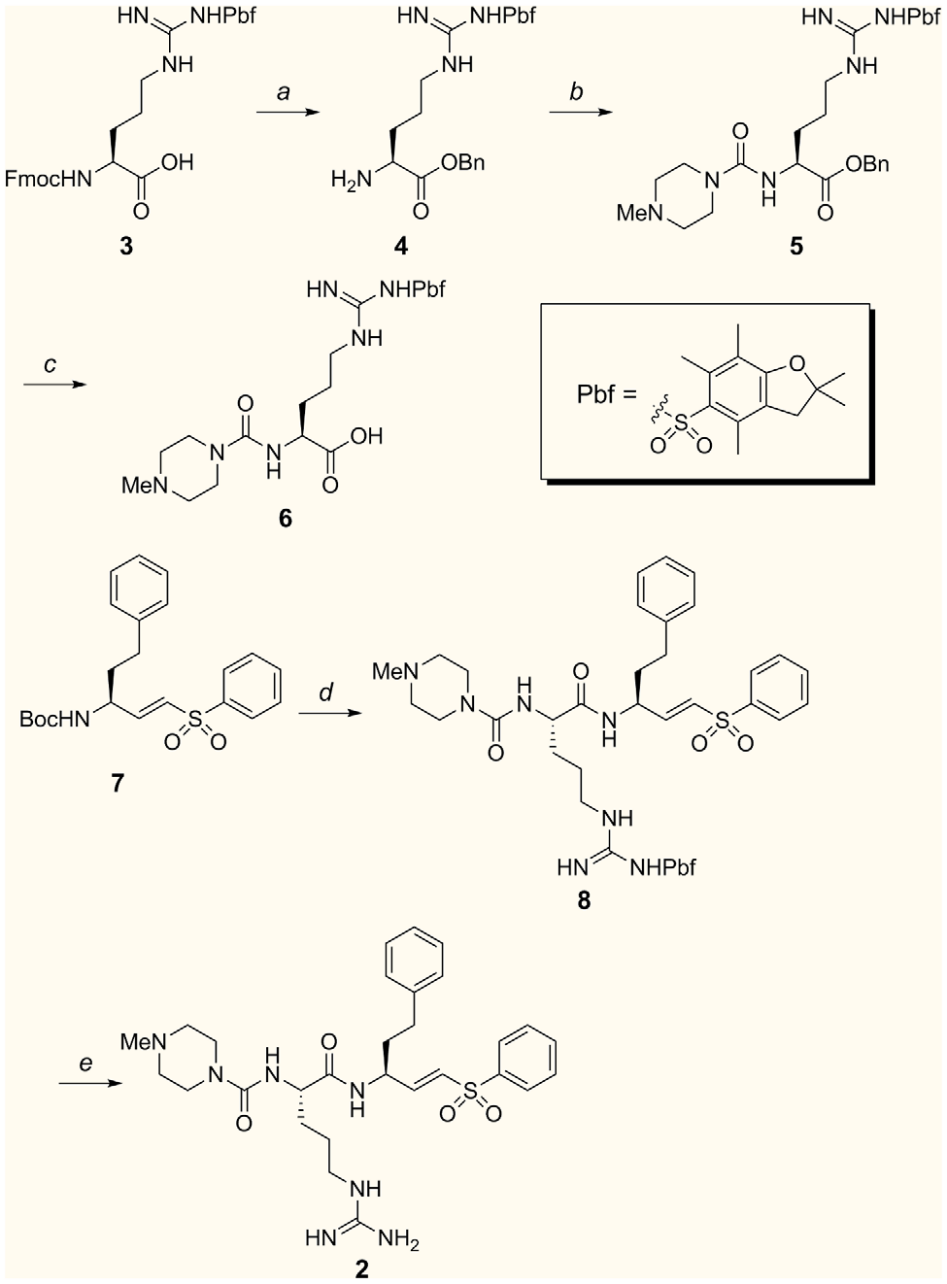
Synthesis of WRR-483 (2): (a) (i) benzyl alcohol (BnOH), 4-dimethylaminopyridine, 1-ethyl-3-(3′-dimethylaminopropyl)carbodiimide hydrochloride (EDC), *N-*methylmorpholine, CH_2_Cl_2_; (ii) piperidine, CH_2_Cl_2_, 90% (two steps); (b) (i) triphosgene, sodium bicarbonate (NaHCO_3_), CH_2_Cl_2_; (ii) *N*-methylpiperazine, CH_2_Cl_2_, 93% (two steps); (c) H_2_, 5% palladium on carbon (Pd/C), MeOH, 92% (d) (i) trifluoroacetic acid (TFA), CH_2_Cl_2_; (ii) **6**, N-hydroxybenzotriazole (HOBT), 1-ethyl-3-(3′-dimethylaminopropyl)carbodiimide hydrochloride (EDC), *N*-methylmorpholine, dimethyl formamide (DMF), CH_2_Cl_2_, 84%; (g) 3:1 TFA:CH_2_Cl_2_, 86%.

The synthesis of WRR-483 is summarized in Figure 2. Esterification of commercially available *Nα*-Fmoc-*Nω*-(2,2,4,6,7-pentamethyldihydrobenzofuran-5-sulfonyl)-L-arginine (**3**) with benzyl alcohol, followed by Fmoc removal gave amine **4**. Amine **4** was converted to an isocyanate by treatment with triphosgene;[38] subsequent addition of *N*-methylpiperazine afforded urea **5**. Deprotection of the carboxylic acid by hydrogenolysis gave the carboxylic acid **6**. The tert-butyl carbonate blocking group of vinyl sulfone **7** [24] was removed and the resulting amine was coupled with acid **6** to give vinyl sulfone **8** in 84% overall yield. Finally, the 2,2,4,6,7-pentamethyldihydrobenzofuran-5-sulfonyl (Pbf) group was removed by treatment of **8** with trifluoroacetic acid in dichloromethane to provide WRR-483 (**2**).

### Biological effects of WRR-483

WRR-483, a K11777 analog, which replaces phenylalanine with arginine at the P_2_ position, was previously reported as an effective inhibitor of the *Entamoeba histolytica* cysteine protease 1 (EhCP1). [39] EhCP1 displays a high preference for arginine at P_2_ that is unusual among the papain-like protease family. In addition, this inhibitor was also demonstrated to successfully reduce amebic invasion in the human colonic xenograft model by 95%.[39] Cruzain is a dual-specific protease that binds to substrates containing phenylalanine or arginine in the P_2_ site, with a preference for phenylalanine over arginine.[13],[18] Hence, we anticipated that WRR-483 would inhibit cruzain, but with lesser efficacy compared to K11777. The inhibitors were also assayed against rhodesain, a closely related enzyme in the protozoan parasite, *T. brucei*. Rhodesain lacks the critical glutamate residue at the S_2_ site for arginine binding, and was reported to be inactive against substrates with arginine at P_2_.[29] Therefore, selectivity for cruzain over rhodesain was anticipated. In the study of irreversible inhibitors, IC_50_ values are highly dependent on assay methods and enzyme concentration, hence the second-order rates of inhibition were determined. As summarized in Table 2, WRR-483 was a modest inhibitor of cruzain, with a *k*_obs_/[I] value of 4,800 s^−1^M^−1^ In comparison, K11777 was more effective than WRR-483 by over 20-fold. As expected, WRR-483 showed no inhibition of rhodesain, even at 10 µM concentration. K11777 and WRR-483 were also assayed against tbcatB, the putative essential protease of *T. brucei*,[30] but both compounds were weak inhibitors.

**Table 2:** Kinetic data for vinyl sulfone inhibitors against cruzain, rhodesain, and tbcatB.

	*k*_obs_/[I] at 1 µM (s^-1^ M^-1^)		tbcatB % inhibition at 1 µM	tbcatB *k*_ass_ (s^-1^ M^-1^)
Cmpd	Cruzain	Rhodesain		
K11777	108,000±4,200	48,000±10,200	48	500±100
WRR-483	4,800±480	No inhibition	26	Not determined

The low rate of inactivation of cruzain by WRR-483 is most likely because cruzain has *ca.* 35% of its maximal activity for arginine containing ligands at pH 5.5.[13],[18] Thus, WRR-483 was re-evaluated in a different buffer condition at a range of pH values. By switching from acetate to a citrate-phosphate buffer, a slight improvement in enzyme activity and inhibitor potency was observed at pH 8.0. The IC_50_ value of WRR-483 showed an almost 10-fold improvement at pH 8.0 than at pH 5.5; however, there was only a 4-fold increase in the second-order rate of inactivation. On the other hand, K11777 showed a consistent IC_50_ value at pH 5.5 and 8.0, while *k*_inact_/[*K*_i_] decreased from 1,030,000 to 234,000 (Table 3).

**Table 3:** pH dependence of cruzain inhibition.

	IC_50_ (nM)		*k*_inact_/*K*_i_ (s^-1^ M^-1^)	
Cmpd	pH 5.5	pH 8.0	pH 5.5	pH 8.0
K11777	1.5±0.8	2±0.3	1,030,000±40,000	234,000±12,000
WRR-483	70±30	8±2	14,000±240	62,000±350

WRR-483 was then tested for its potency on arresting the intracellular growth of *T*. *cruzi* infection in J774 macrophages. The effectiveness of the inhibitor was determined by the number of days the lifetime of the treated infected cells were prolonged compared to the control (Table 4). In the absence of the inhibitor, parasite-infected macrophage controls lysed in six days, due to the completion of the parasite intracellular replication cycle. Surprisingly, at 10 µM, WRR-483 was as effective as K11777 in eliminating the parasite from the host cells, as the cultures with the vinyl sulfones stayed intact until the experiment was terminated. Even after inhibitor treatment was ceased on the 27^th^ day, no re-emergence of parasite infection was observed for two weeks thereafter, at which point the experiment ended. Determination of dose response of the activity of WRR-483 against *T. cruzi* infection in BESM cells demonstrated that WRR-483 had an EC_50_ of 0.2 µM, which was more effective than K11777 and comparable to posaconazole (EC_50_ = 4.2 and 0.2 µM for K11777 and posaconazole, respectively).[34]

**Table 4:** Results of inhibitors on survival of J774 cells and mice infected by *T. cruzi*.

Compound	*T. cruzi* infected cell survival (days) a	*T. cruzi* infected mouse survival (days)^b^
Control	6	21
K11777	47	110
WRR-483	47	110

*^a^*J774 macrophages infected with *T. cruzi* were treated every 48 h until Day 27 with a solution of inhibitor (10 µM). Survival time is defined as the number of days before the cell monolayer is destroyed by the infection. Experiment was terminated on Day 47.

*^b^*Infected mice were treated with 100 mg inhibitor/kg body weight in two daily doses.

### Murine model studies

Encouraged by the anti-parasitic activity in the macrophage assay, WRR-483 was further evaluated *in vivo*. In a murine model of acute Chagas' disease, mice were exposed to 10^6^ trypomastigotes of the virulent CA-I/72 clone (Table 5). All untreated control mice infected with *T. cruzi* died within 21–60 days while all mice treated with WRR-438 or K11777 survived the acute infection and appeared healthy and normal. Tissues inspected histologically including heart and skeletal muscle were normal and free of parasites in 3/5 mice treated with WRR-483 and 3/5 mice treated with K11777; 2/5 mice in each group had 1–3 nests of amastigotes visible in skeletal muscle while heart tissue was normal or presented mild inflammatory infiltrate. In contrast, 5/5 untreated controls had *T. cruzi* amastigotes and intense or moderate inflammation in heart and/or skeletal muscle. Hemocultures were negative for all inhibitor treated mice and positive for untreated controls.

**Table 5:** Histopathology of mice infected by T. cruzi.

Treatment	Animal ID#	Heart	Sk. muscle	Liver	Spleen	Colon
Untreated controls	307-1-1	+A i/i	+10A Intense i/i Necrosis	N	N	N
	307-1-2	i/i	+A i/i	N	N	N
	307-1-3	i/i	+A i/i	N	N	N
	307-1-4	i/i	+A i/i	N	N	N
	307-1-5	i/i	+AIntense i/i	N	N	N
WRR-483	307-2-1	Mild i/i	+AIntense i/i	N	N	N
	307-2-2	N	+AIntense i/i	N	N	N
	307-3-3	N	Moderate i/i	N	N	N
	307-3-4	N	Moderate i/i	N	N	N
	307-3-5	N	Moderate i/i	N	N	N
K11777	307-5-1	Mild i/i	+AIntense i/i	N	N	N
	307-5-2	N	Moderate i/i	N	N	N
	307-5-3	N	+AModerate i/i	N	N	N
	307-5-4	N	Moderate i/i	N	N	N
	307-5-5	N	Intense i/i	N	N	N

I/I: Inflammation and infiltration.

+10A: Numerous nests of *T. cruzi* amastigotes.

+A: 1-3 nests of *T. cruzi* amastigotes.

N: Negative.

### Protease inhibition profiling

To identify any possible off-target activity, and to assess selectivity, WRR-483 and K11777 were screened against a panel of 70 proteases (Reaction Biology Corp). The proteases that WRR-483 and K11777 inhibited with IC_50_ values less than 10 µM are shown in Table 6. The inhibitors were active against only papain and a few of the papain-like members of the cathepsin family, particularly cathepsins B, C, and S. However, potency of WRR-483 against papain, cathepsins L and V were moderate, and no inhibition of other cysteine proteases, including calpain-1, cathepsins H and K, was observed. Overall, the selectivity profile of WRR-483 was comparable to K11777, but WRR-483 had a lower affinity for all proteases except for cathepsin B.

**Table 6:** Inhibition of other proteases by K11777 and WRR-483.

	IC_50_ (nM)	
Protease	K11777	WRR-483
Cathepsin B	5.7	3.9±0.1
Cathepsin C	0.4	7.0±0.9
Cathepsin K	25	No inhibition
Cathepsin L	0.2	53±4
Cathepsin S	0.6	1.4±0.01
Cathepsin V	1.2	335±4
Papain	0.4	373±44

### Structure determination

**Figure 3:**
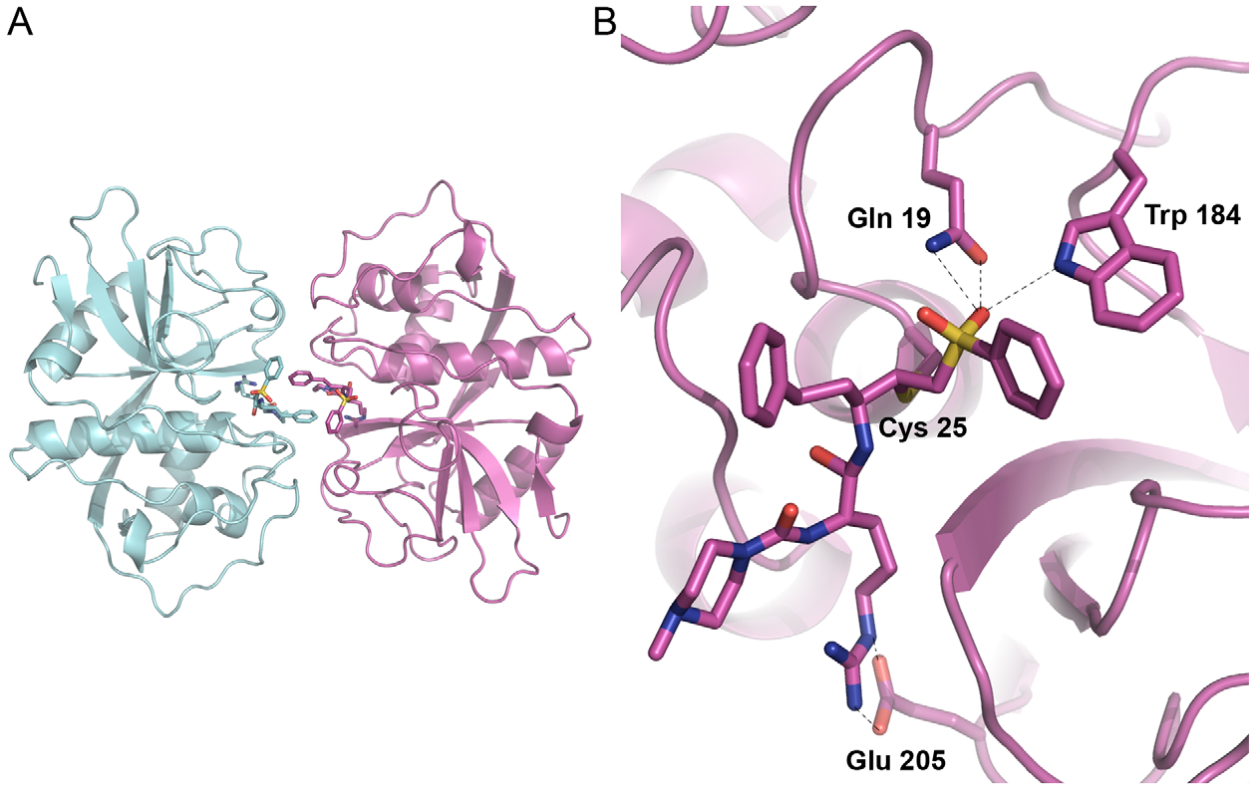
Structure of WRR-483 bound to cruzain . (A) Crystallographic unit of the WRR-483-cruzain complex. (B) View of the active site of cruzain. The catalytic Cys25 and residues involved in binding to the inhibitor are also shown. Figures prepared with PyMol.

A 1.5 Å crystallographic structure of cruzain with WRR-483 was determined. Two unique complexes (A and C) of cruzain covalently bound to the inhibitor WRR-483 comprise the crystallographic unit (Figure 3). The two cruzain molecules are nearly identical in structure, with a RMS distance of 0.17 Å when superimposed upon one another. The two inhibitor molecules are also nearly identical in conformation and placement within the active site of cruzain, with the exception of the conformation of the N-methyl-piperazine group at the P_3_ position. The packing of the asymmetric unit reveals that the opening of the active site cleft of each cruzain molecule points toward the other, such that the two WRR-483 molecules within the asymmetric unit are directly adjacent to one another. The homophenylalanine moiety of the inhibitor molecules come within a 3.6 Å distance of one another at the point of closest proximity. This packing appears to be an artifact of crystal packing in this space group, with these crystallization conditions.

The WRR-483 inhibitor molecule is covalently bound to the active site cysteine *via* Michael addition, at a distance of 1.84 Å in complex A and 1.83 in complex C (Figure 3b). Other points of contact between cruzain and the inhibitor are fairly consistent between complex A and C. The vinyl sulfone moiety makes several constructive interactions to the protein. One of the vinyl sulfone oxygens forms hydrogen bonds with His162 Nδ1 (3.39 Å in complex A, 3.43 Å in complex C), Gln19 Nε2 (3.09, 2.90 Å) and with Gln 19 Oε1 (2.94 Å, complex A) while the other oxygen makes a 2.98 Å hydrogen bond to an ethylene glycol molecule in complex A. This nexus of interactions is consistent with what was observed in several other X-ray crystal structures of cruzain with bound vinyl sulfone inhibitors.[14],[16] When the cruzain-WRR-483 structure is superimposed on several other structures of cruzain bound to vinyl-sulfonyl containing inhibitors (PDB 1F2A, 1F2B, 1F2C, 2OZ2), the positioning and orientation of the vinyl sulfone moieties are nearly perfectly aligned (data not shown).

Hydrogen bonding interactions between cruzain and the peptide backbone of WRR-483 are consistent with the previously reported complexes. These include hydrogen bonding between the peptidyl oxygen of Asp161 and the amide nitrogen of the inhibitor (2.89, 2.86 Å), the peptidyl nitrogen of Gly66 with the amide oxygen of WRR-483 (3.00, 3.02 Å), and the peptidyl oxygen of Gly66 with the urea nitrogen of WRR-483 (2.85, 2.91 Å). Hydrogen bonding interactions are also formed between the urea carbonyl oxygen of the inhibitor and two water molecules in complex A at distances of 2.83 Å and 3.36 Å. In complex C, there is one such interaction at this point, at a distance of 2.83 Å. Like the other vinyl sulfone inhibitors, the homophenylalanine side-chain extends into the solvent without making any favorable contacts with the enzyme.

The most interesting of interactions, because they are located within the bottom of the S_2_ pocket, the site known to impact substrate preference profiles for papain-family cysteine proteases, are with the Arg moiety of WRR-483. The Nε of the Arg moiety of WRR-483 makes contacts with the Oε2 of Glu208 at distances of 2.84 Å and 2.81 Å in the two complexes. The Nη of the inhibitor arginine moiety binds to the Oε1 of Glu208, at distances of 2.89 and 2.89 Å. The remaining hydrogen bond contacts with the arginine group of the inhibitor are to the amide nitrogen. In complex A, there is a hydrogen bond formed to a water molecule at a distance of 2.99 Å.

## Discussion

Kinetic studies indicate that WRR-483 is a modest inhibitor of cruzain. This compound is more potent at higher pH levels, but it still relatively weak when compared to K11777. Yet, in the *in vitro* cell assay, this compound demonstrates trypanocidal activity comparable to the lead compound, K11777, and is unexpectedly effective in curing acute *T. cruzi* infection in mice. Crystallographic evidence indicates that WRR-483 indeed binds to cruzain in a similar fashion compared to other vinyl sulfone analogs, with the addition of hydrogen bonding interactions between Glu208 in the S_2_ pocket and the arginine side chain of the inhibitor. The structure of cruzain bound to another irreversible inhibitor containing an arginine in the P_2_ position has been solved previously.[13] In this structure, two conformations of the arginine side chain of Z-Arg-Ala-FMK have been modeled at partial occupancies, with the one identified as more physiologically relevant at *ca.* 70% occupancy (PDB ID 2AIM). In the major conformation, the NH1 and NH2 of the arginine moiety form a substrate-directed salt bridge to the glutamate in the base of the S_2_ pocket. The glutamate (Glu 205) side chain is oriented such that it points into the S_2_ pocket, towards the guanidine group. This is in contrast to how this residue is often found, swung out towards solvent, in structures that contain a hydrophobic P_2_ moiety which is not conducive to forming a salt bridge, hydrogen bond or another constructive electrostatic contact.[10],[12]

**Figure 4:**
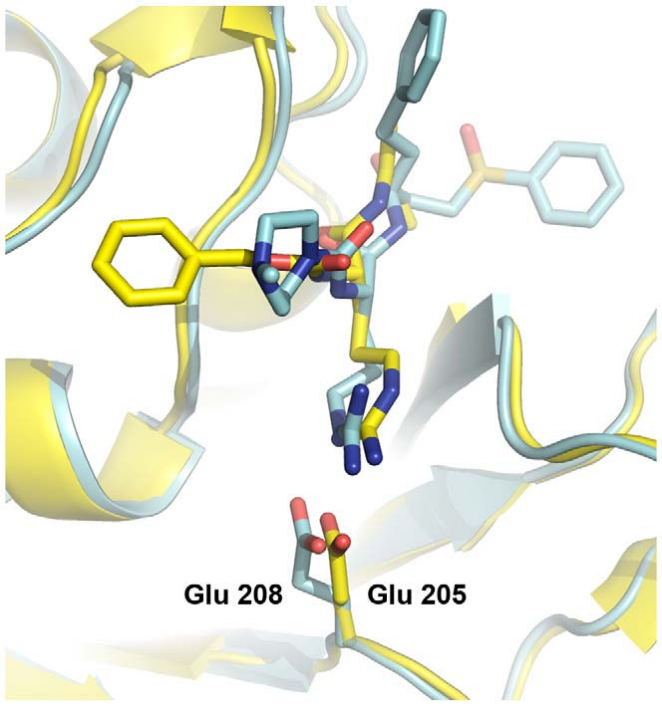
Comparison of the binding modes of WRR-483 and Z-RA-FMK. Superimposition of the cruzain-WRR-483 structure (blue) on cruzain-Z-RA-FMK (yellow). Bound inhibitors are colored as their respective cruzain model. The glutamate residue in the S_2_ pocket which binds to the guanidine moiety of the inhibitor (Glu205 and 208 for Z-RA-FMK and WRR-483, respectively) are highlighted. Figure prepared with PyMol.

The overall structure of 2AIM and the currently described structure are globally similar. Superimposition of the two structures results in an RMS distance of 0.469 Å. However, the two structures have distinct interactions between the P_2_ arginine and the S_2_ glutamate. While the arginine side chains are similarly oriented at the Cβ and Cγ positions, Cδ is positioned differently, as a result of a rotation of *ca.* 180° about the Cβ-Cγ bond. This results in a subsequently different positioning of the remaining atoms in the moiety. The arginine Nε of WRR-483 sits approximately where NH1 of the Z-Arg-Ala-FMK was positioned, and one terminal nitrogen of WRR-483 is approximately in the same position as the corresponding nitrogen of the FMK inhibitor. The other terminal nitrogen of WRR-483 is directed towards solvent (Figure 4). The conformation of the S_2_ glutamate is also different between the two structures. While still largely directed into the S_2_ pocket, Glu208 in cruzain-WRR-483 is less fully anchored to the arginine and therefore is not as well contained.

There are several interpretations for the rather unexpected anti-parasitic properties of WRR-483. It is conceivable that WRR-483 is inhibiting more than one parasitic protease. Other potential targets include the cathepsin B-like protease[40] and cruzipain-2, an isoform of cruzain which was reported to be active against substrates containing an arginine group in the P_2_ position.[41] Protease profiling studies indicate that WRR-483 is only active against a few members of the cathepsin family. Also, we have previously demonstrated that WRR-483 is a potent inhibitor of the cathepsin L-like cysteine protease, EhCP1, of *E. histolytica*, and is much more effective against EhCP1 than K11777. [39] Thus, it is possible that WRR-483 is targeting an as yet unidentified cathepsin L-like cysteine protease in *T. cruzi* as well.

Another possibility is that the WRR-483 inhibits cruzain either located on the cell membrane or released by the parasite, and not in the lysosome. The inhibitor is hydrophilic in nature, primarily due to the guanidine group, and is more active at physiological pH, making it very favorable in the extracellular environment. Parasite cysteine proteases function in a broader pH profile than their host orthologues, which makes them more susceptible to the inhibitor. As a part of the host cell invasion mechanism, cruzain is secreted by *T. cruzi* trypomastigote to release an invasion factor from the parasite membrane.[42] Selective inhibition of the released extracellular cruzain, in effect, could lead to inhibition of host invasion. This was also observed in the study of invasion by *E. histolytica*. In this case, WRR-483 was found to be effective in reducing host invasion by inhibiting the cysteine proteasethat is released by the organism.[39]

In this study, we have identified WRR-483, as an effective agent to treat acute Chagas' disease in a murine model. This compound was as effective as the lead compound, K11777, in eliminating parasite proliferation despite displaying modest potency against cruzain. The crystal structure of WRR-483 complexed to cruzain was solved and established the binding mode of the inhibitor to the enzyme. This structure was highlighted by the formation of a salt bridge between the guanidine moiety of the inhibitor and Glu208, but in a different conformation when compared to another structure of cruzain bound to a different arginine-containing inhibitor. WRR-483 has recently completed pharmacokinetic and toxicology studies (SRI international) and was shown to possess a decent pharmacokinetic profile (*Cl* = 27.5 mL/min/kg, *V*_d_ = 15.1 L/kg, *t*_1/2_ = 6.4 h at 10 mg dose iv) but low oral bioavailability due to the polar arginine residue (%*F*<1%). The no observed adverse effect level (NOAEL) is higher than 100 mg/kg. These data suggest that WRR-483 may be useful for treating parasitic infections like *T. cruzi* as an IV agent, or *E. histolytica* infections of the intestinal tract. Further studies to understand the precise mechanism of trypanocidal action of WRR-483 and design of analogs with improved bioavailability are currently underway.
